# Assessing the Potential for Genetic Rescue in Critically Endangered Saimaa Salmon: Early Viability and Genomic Outcomes via First Backcrossing

**DOI:** 10.1111/eva.70349

**Published:** 2026-11-01

**Authors:** Matti Janhunen, Miika Tapio, Aslak Eronen, Raine Kortet, Antti Kause

**Affiliations:** ^1^ Natural Resources Institute Finland (Luke) Joensuu Finland; ^2^ Natural Resources Institute Finland (Luke) Jokioinen Finland; ^3^ Department of Environmental and Biological Sciences University of Eastern Finland Joensuu Finland

**Keywords:** conservation breeding, genetic rescue, genomic diversity, hybridization and backcrossing, inbreeding depression, Saimaa salmon

## Abstract

Small, threatened populations often suffer from a limited gene pool, which increases inbreeding and reduces their adaptability. We investigated the phenotypic and genomic effects of hybridization (resulting from backcrossing) on the critically endangered Lake Saimaa landlocked salmon, *Salmo salar* m. *sebago*, focusing on a particularly sensitive alevin‐fry life stage. The Saimaa salmon has undergone severe genetic bottlenecks and relies exclusively on a hatchery‐based conservation program. Since 2017, crossings with geographically proximate anadromous Baltic Sea salmon populations (Neva and Tornio) have been conducted as a possible means to restore genetic diversity. Here, we used a maternal half‐sib design to compare early viability of purebred Saimaa salmon with their first‐generation Neva and Tornio backcrosses in semi‐natural conditions. The backcrossed fry survived better than purebreds, showing a 9–12 percentage‐point increase in viability, whereas no significant cross differences were found in body mass. Quantitative genetic analysis further showed that hybrid sires conferred higher genetic merit for survival. Genomic analyses using a high‐density SNP array (47,901 loci) confirmed clear differentiation between pure and backcross groups, validating admixture. Pure Saimaa salmon had heterozygosity (*H*_S_) of 0.13 (over 50% of loci monomorphic), while backcrosses possessed *H*_S_ of 0.25 and substantially more polymorphism (96%–97% of loci). Analysis of homozygous genomic segments showed that the inbreeding of Saimaa salmon has predominantly accumulated prior to the conservation program. Widespread reductions in autozygosity were found in both cross types, though introgression varied across the genome and also showed substantial donor‐ and family‐specificity. Overall, the results suggest that hybridization improved early survival through additive and potential dominance effects (reduced genetic load, heterozygote advantage), and expanded genomic variation without immediate indication of outbreeding depression. However, sequential backcrossing across multiple generations is necessary to ensure the preservation of Saimaa salmon's unique genetic identity while enhancing its resilience through the life cycle.

## 1 Introduction

Small and isolated populations are typically characterized by low genetic diversity (Frankham 1997; White and Searle 2007; Jensen et al. 2013; Subramanian and Kumar 2023), and their evolutionary trajectories are strongly influenced by random genetic drift (Woolfit and Bromham 2005). This process, together with an increased accumulation of homozygous and potentially deleterious alleles, may reduce the adaptation potential of a population or even elevate extinction risk (Saccheri et al. 1998; Keller and Waller 2002; Robinson et al. 2019). For endangered populations or species with the absence of natural gene flow, reduced genetic diversity can be restored, and the harmful effects of inbreeding counteracted, by introducing genetic resources from external populations (i.e., genetic rescue; Kristensen et al. 2015; Whiteley et al. 2015; Ralls et al. 2018; White et al. 2023; Clarke et al. 2024). Such assisted gene flow, however, presents a potential drawback: in some cases the admixture of genetically divergent populations involves the loss of local adaptations, resulting in a reduction in fitness known as outbreeding depression (Tallmon et al. 2004). Hybridization is also commonly seen as harmful because it can lead to genetic swamping (i.e., gradual replacement of the original gene pool by a foreign one) and loss of population or species identity (Todesco et al. 2016; Adavoudi and Pilot 2022).

In many fishes, including salmonids, high fecundity and external fertilization permit effective and controllable management of stocks from different origins through hatchery propagation, and hence brood fish from distinct populations can be crossed artificially in vitro (Burridge 2019). Unlike the direct translocations of foreign individuals, the use of controlled crosses in aquaculture ensures that new genetic variation is incorporated into the recipient population. In Finland, the potential of hybridization is being evaluated for the critically endangered and genetically impoverished landlocked Atlantic salmon ( *Salmo salar* m. *sebago*), endemic to the Vuoksi watercourse and its principal basin, Lake Saimaa (Hutchings et al. 2019). The population collapsed by the early 1970s, when the last natural breeding grounds in River Pielisjoki were destroyed by canalization and damming of the river for hydropower production (Pursiainen et al. 1998; Eronen et al. 2021). The present Saimaa salmon population descends from the last few wild founders captured during the years 1963–1969. Since then, the existence of the Saimaa salmon in its original range has been entirely based on supportive breeding and annual stockings of hatchery‐reared juveniles, mainly 2‐year‐old smolts (Pursiainen et al. 1998; Janhunen et al. 2023, 2025). Since 1975, the statutory conservation program has annually renewed the captive broodstock with wild‐caught spawners that have undergone a partial life cycle in the wild (feeding migration in lakes Saimaa and Pielinen) (for more information, see Eronen et al. 2023; Janhunen et al. 2023; Eronen 2024).

To test hybridization as a potential supplementary conservation action, crosses have been made since 2017 between the Saimaa salmon and two geographically close anadromous (Baltic Sea) salmon populations originally spawning in rivers Neva and Tornio (Figure 1) (Eronen et al. 2021, 2023). Previously reported heterozygosity levels of these anadromous salmon populations, based primarily on microsatellite data, are significantly higher (0.60–0.75) compared to the Saimaa salmon (0.28–0.47) (Koljonen et al. 2002; Tonteri et al. 2005). Based on phylogenetic studies and the knowledge of post‐glacial geological phases (Nilsson et al. 2001; Tikkanen 2002; Säisä et al. 2005; Lumme et al. 2016), the Saimaa salmon may have been separated from its nearest Baltic Sea conspecifics for around 10,000 years. While the higher genetic diversity of the donor populations supports their use for the genetic rescue of Saimaa salmon, the long‐term isolation of the populations raises concerns about outbreeding depression.

**FIGURE 1:**
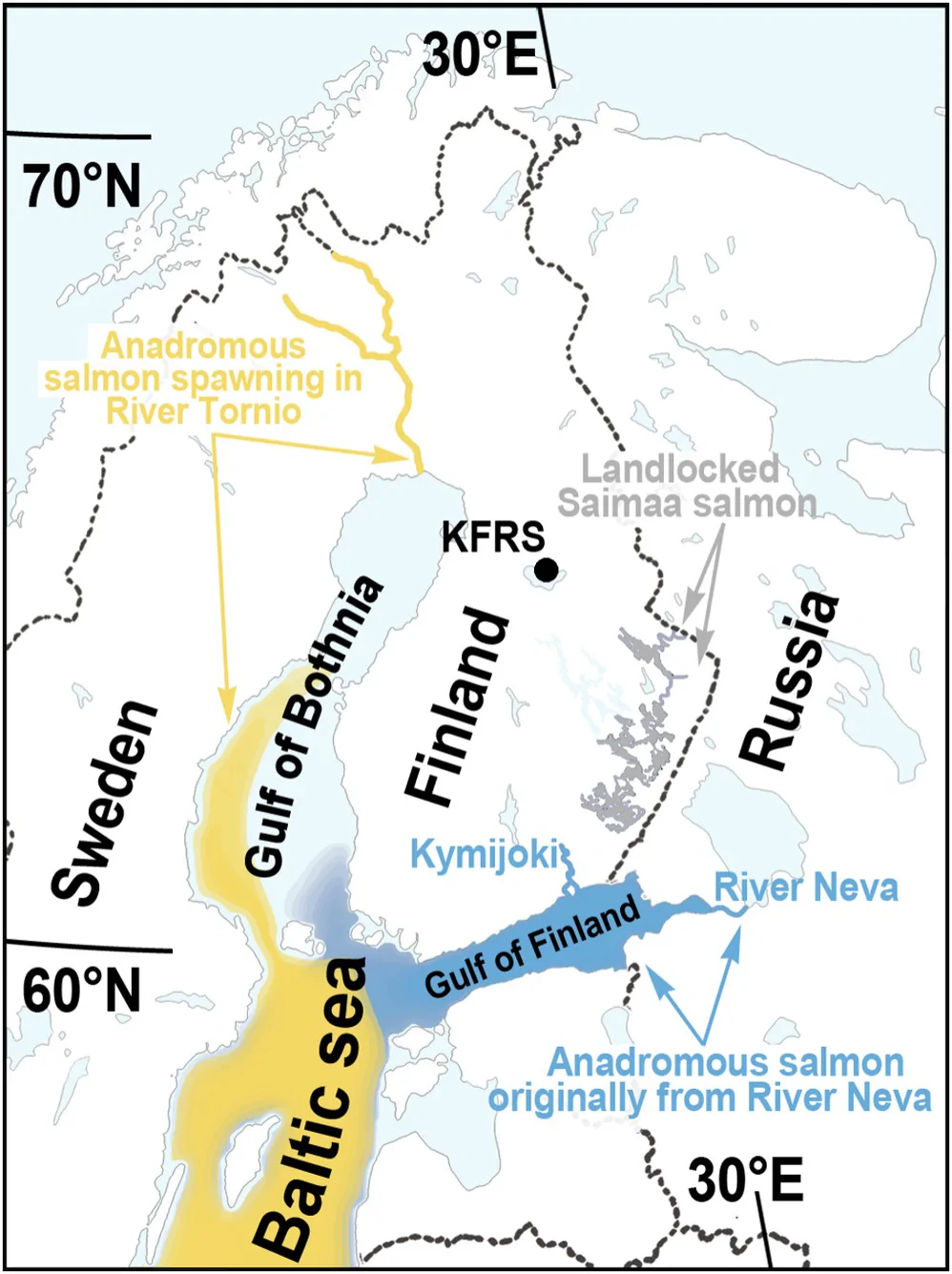
A map showing the natural distributions of the hybridized salmon populations, and the location of study site (Kainuu Fisheries Research Station, KFRS). Original distribution of landlocked Saimaa salmon is shown in gray: River Pielisjoki—Lake Saimaa system and River Lieksanjoki—Lake Pielinen system (Seppovaara 1962; Pursiainen et al. 1998). Blue color indicates the range of anadromous Neva salmon: River Neva, where the population originates from, River Kymijoki, where natural reproduction occurs as a result of stocking (reintroduction in 1979), and the approximate feeding area of the population in the Baltic Sea (Kallio‐Nyberg and Ikonen 1992). Correspondingly, yellow color shows the main natal river and approximate feeding area of the anadromous Tornio salmon. The map was drawn by hand using a base map from Google Maps 2020 (edited in Adobe Photoshop CS4; Berkeley, California, Peachpit Press).

Experimental comparisons between purebred Saimaa salmon and first‐generation hybrids (F1) have so far revealed both positive and negative effects of hybridization on performance traits. Eronen et al. (2021) found, for example, no obvious prezygotic constraints (incompatibility effects) due to hybridization, and further, the hatching success and early post‐hatching survival under semi‐natural stream conditions were even improved by crossbreeding. In another study, juvenile hybrids showed better avoidance of a piscine predator (Northern pike, *Esox lucius* ) than the pure Saimaa strain, but this apparent fitness benefit was offset by a 12%–23% increase in eye cataracts caused by a common trematode eye parasite (*Diplostomum pseudospathaceum*) (Eronen et al. 2024). The better *Diplostomum* resistance of Saimaa salmon, compared to the anadromous populations, likely results from stronger selection pressure (coevolution) in the freshwater environment, where the landlocked population is exposed to the parasite throughout its life cycle. Hence, the observed reduction in resistance due to hybridization can be clearly seen as a sign of outbreeding depression, at least in the F1 phase (Klemme et al. 2021, 2024).

Considering the long‐term isolation and severe population bottleneck(s), even a small increase in genetic diversity might promote the resilience and adaptive potential of the Saimaa salmon. The aim in producing the hybrid strain for possible stock management is to preserve the high genetic share (uniqueness) of the original Saimaa population. Therefore, a hybridization program requires repeated backcrossing of at least three generations (until F4 or more), when the proportion of foreign genome is gradually diluted to a few percentages (Graf 2024). However, some signs of outbreeding depression may only appear in the second or later hybrid generations, where recessive incompatibilities are exposed in homozygous genotypes and (or) recombination breaks up locally adapted epistatic gene combinations (Wright 1931; Edmands 1999, 2007; Gharrett et al. 1999; Tallmon et al. 2004; Goldberg et al. 2005; Whitlock et al. 2013). A comprehensive risk assessment thus needs to be extended over two or more generations before further conclusions about the suitability of hybridization can be made and before any such fish material can be introduced in the original range of the Saimaa salmon (Edmands 2007).

In addition to phenotypic assessment, monitoring changes in genetic diversity is an essential part of assisted gene flow (Robinson et al. 2017; Theissinger et al. 2023; Schmidt et al. 2024; Kobayashi et al. 2025). Although the conceptual value of genetic rescue interventions is well recognized, empirical applications—especially using genomic information—remain rare in conservation breeding programs of fishes (Supple and Shapiro 2018). On the other hand, advanced genomic resources, such as single‐nucleotide polymorphism (SNP) arrays covering the whole genome, are already available at reasonable cost for many economically important species, such as the Atlantic salmon. These tools not only allow monitoring and predicting the genomic consequences of hybridization, but they can also be effectively used to design breeding strategies, identify variation between and within populations for key traits under selection, and to assess potential trade‐offs between inbreeding and outbreeding depression (Bell et al. 2025). Genome‐wide SNP data can also be used to identify runs of homozygosity and homozygous‐by‐descent segments, providing genomic measures of realized autozygosity or inbreeding that are less dependent on allele‐frequency estimates (McQuillan et al. 2008; Curik et al. 2014).

This experimental study provides the first integrated assessment combining phenotypic data on early performance with information on genome‐wide variation in the Saimaa salmon and their maternal backcrossed half‐sib families. After a one‐month rearing period outdoors, simulating natural stream conditions, we first examined whether the purebred Saimaa salmon and two backcrosses (Tornio and Neva) differed in survival and growth, and further compared the mean genetic merits for the traits between sires of different genetic backgrounds (pure and two hybrid groups). Second, using a high‐density SNP panel, we analysed the genomic diversity within and among the crossing groups. Among the first studies in fish (Yoshida et al. 2020), we evaluated the impact of hybridization (admixture) on genomic diversity through population indices, such as realized inbreeding, effective population size (*N*_e_), and specifically examined how backcrossing affected the long runs of homozygous genomic segments (ROH‐like regions; McQuillan et al. 2008; Ceballos et al. 2018; Zhang et al. 2015) by introducing new allelic variation into previously identical‐by‐descent blocks.

## 2 Materials and Methods

### 2.1 Gamete Collection, Fertilizations, and Incubation

The comparison of purebred and backcrossed salmon groups was performed using a maternal half‐sib family design, allowing us to examine phenotypic and genetic influences due to paternal source of variation. The gametes of Saimaa salmon were collected from 19 females (13 Oct 2022; year class 2015) and 19 males (14 Oct 2022; year class 2019) maintained in a captive broodstock at Taivalkoski Aquaculture Station by the Natural Resources Institute Finland (Luke). In order to avoid direct inbreeding within the Saimaa salmon, the female and male parents were of different year‐classes; a standard practice in captive breeding of the Saimaa salmon. The year class of Saimaa female parents descended from 59 wild‐caught females and 9 wild‐caught +13 hatchery‐reared males (effective population size, calculated directly from the number of females and males; Wright 1931: *N*_e_ = 64), and the year class of the male parents descended from 100 females and 70 males (all wild‐caught, *N*_e_ = 164). To better include the within‐population genetic diversity in the experimental families, the Saimaa salmon females were selected based on their expected genetic relationships that were determined earlier as part of the broodstock management using 15 microsatellite markers. Thus, 11% (17/154) of all pairwise relationships among the females were recognized as half‐siblings (average 1.9 and max. 3 half siblings per female), and the rest were genetically more distant. Stripped eggs were stored in 0.5 L plastic airtight containers and stripped milt in 0.5 L plastic ziplock bags filled with pure oxygen. All the gametes were stored on ice and transferred to the Kainuu Fisheries Research Station (KFRS) in Paltamo to await fertilizations. The milt bags were refilled with pure oxygen every 2–3 days before the fertilizations.

Milt from F1 hybrid salmon males (year class 2018) were collected from the hatchery broodstock reared at KFRS (19 Oct 2022). The establishment of the F1 hybrid generation is described by Eronen et al. (2021). There were 11 Saimaa × Neva males, 8 Neva × Saimaa males, 9 Saimaa × Tornio males, and 10 Tornio × Saimaa males (maternal strain given first). For balanced statistical comparisons, however, only two backcrossed groups with different anadromous population backgrounds were used (abbreviated hereafter as BC_Neva_ and BC_Tornio_), regardless of whether the hybrid sires descended from a landlocked or anadromous dam/sire.

The in vitro fertilizations were conducted on 20 Oct 2022 using a maternal nested mating design, where each female was mated with a purebred Saimaa male and two F1 hybrid males. Each male was used to fertilize only one female's eggs. Hence, the total number of matings was 57 (19 × 3 maternal half‐sib groups), and each full‐sib family was further replicated three times, forming 171 fertilizations (egg batches) in total. The eggs from each female were split into nine portions using roughly similar numbers of eggs per batch (mean 98, range 89–104 eggs, *n* = 171 egg batches), which was determined based on female‐specific mean egg mass (weighted sample of eggs without ovarian fluid). The fertilizations were performed in 0.2 L plastic cups by pipetting 200 μL of milt directly onto the eggs and then immediately activating the sperm with water. For some hybrid males, only 50–100 μL of milt was used per egg batch due to small milt dose available. However, also this smaller volume of milt is considered more than enough to fertilize a relatively low number of eggs of the batch.

Following fertilizations, the egg batches were let to swell for a few minutes in the fertilization cups and then moved to separate plastic incubation cylinders (ø 10.2 cm) with a net bottom (mesh size 3 mm) (see Eronen et al. 2021; Janhunen et al. 2024). The 171 floating cylinders circled slowly in groups of eight units in 23 round 0.4 m^2^ tanks, except for one tank which only contained three incubation units. The cylinders were randomized among the tanks. Incubation temperature (range 1.0°C–11.9°C) and oxygen content (range 6.3–11.6 mg L^−1^) followed natural variation of the inflowing water from Lake Kivesjärvi (22 Oct 2022–27 May 2023). To prevent the occurrence and spread of oomycete (*Saprolegnia* sp.) infections, dead eggs and alevins were counted and removed from cylinders weekly. To identify fertilized eggs at the eyed‐embryo egg stage (20–24 Feb 2023, heat sum 242–249 degree‐days °C), embryos were mechanically disturbed by pouring each egg batch into a plastic cup. Thereafter, the eggs that were unfertilized or contained an aborted embryo turned white and were removed (mean ± SD survival = 54.2% ± 27.2%, range 0.0–93.1, *n* = 57 families).

Before hatching, all egg batches were moved into cylinders with a smaller mesh net (1 mm) to prevent alevins from escaping (29–30 Mar 2022). Then, 105 cylinders with more than 50 eggs were further divided into two or three replicates to avoid overcrowding and the consequent blockage of water. The embryos hatched in April 2023.

### 2.2 Comparison of Crosses in Post‐Hatching Experiment

The experiment to compare survival and growth of crosses from late alevin (swim‐up) stage until the fry stage was initiated on 27 May 2023. Then, samples of alevins were collected (524 degree‐days °C from fertilizations) from 18 × 3 maternal half‐sib groups (54 full‐sib families) that had enough hatched alevins alive for two replicate batches. The initial number of alevins was equalized to 20 per family and their replicate. Using a similar approach as Eronen et al. (2021), these alevin batches were moved to 0.24 m^2^ flow‐through boxes (40 × 60 cm, front and back walls partially replaced with a 2 mm mesh‐size net), which contained a gravel bottom (~30–80 mm grain size, water depth 15–20 cm) and were placed into eight circular semi‐natural outdoor stream channels (outer circumference 31.0 m, inner circumference 21.4 m, width 1.5 m). There were 14 boxes within each experimental channel, 13 or 14 of which contained fish. The family‐specific boxes were positioned into seven rows of two boxes evenly distributed in each channel, and the full‐sib family replicates were always assigned to different channels. The total number of alevins within the 54 families was 2159 at the beginning of the experiment. The fish were not fed during the experiment (31–32 days), but they were dependent on natural live prey drifting into the boxes with water flow. To ensure sufficient flow of water within the boxes, their front and back meshes were cleaned twice a day using a long‐handled brush.

Survival to fry stage was recorded as the proportion of individuals that were alive at the end of the experiment in each box (27–28 Jun 2023). To compare growth of the crosses, final total length (accuracy 1 mm; mean ± SD = 30.7 ± 2.0 mm, min–max = 25–37 mm) and fresh body mass (accuracy 0.001 g; mean ± SD = 2.203 ± 0.539 g, min–max = 0.953–4.064 g) were recorded for samples of fry from each experimental family (*n* = 9–13 fish per family and 644 fish in total). The size measurements were performed in three consecutive days: the first group of 40 live fry were sampled on 27 Jun. These fish were inspected for possible background infection of *Diplostomum* eye fluke prior to experimental infections (see below) and represented 22 full‐sib families sampled from each experimental channel and from 4 to 5 boxes per channel (*n* = 1–3 fry per family). On the following day (28 Jun), some of the survivors were sampled equally from all three families of 10 females (*n* = 11–12 fry in 30 full‐sib groups) for experimental *Diplostomum* infections; results from that trial not presented here. At the same time, samples of 7–12 fry (*n* = 245 fish in total) were measured for the remaining 24 families that were not used in infections. The infection period lasted over one night, during which the infected fish were kept in family‐specific containers used in egg incubation. This group of fish were measured on 29 Jun (*n* = 359 fry in total). Infection treatment had caused the death of 178 fry (49.6% of all infected fish), but these fish were also measured together with survivors. All live fish were sacrificed with an overdose of benzocaine before the size measurements, and a subset of 4–6 fry from each family was stored in 99% ethanol (Individual 1.5 mL Eppendorf tubes) for later DNA extraction and genomic analysis. Because the phenotypic correlation between length and body mass was high (Pearson's *r* = 0.900, *p* < 0.001, *n* = 644 fish), only body mass was used in data analyses.

The study complied with the animal care legislation of Finland. Experimental infections of the fish with *Diplostomum* sp. were performed with permission issued by the Finnish Regional State Administrative Agency (National Animal Experiment Board: license no. ESAVI/8397/2021).

### 2.3 Genomic Data Collection

Genomic DNA was extracted from 97, 95, and 95 individuals from Saimaa, Neva backcross to Saimaa (BC_Neva_) and Tornio backcross to Saimaa (BC_Tornio_) groups, respectively. To be able to quantify the overall genomic diversity of the crosses as well as the regions into which new genomic variation is introduced by backcrossing, the samples were genotyped using a High Density (70 k) Atlantic Salmon Axiom SNP array (Benchmark Genetics, Bergen, Norway) covering all chromosomes. The initial genotype dataset comprised 68,497 SNPs. For quality control, markers were excluded if the call rate was below 99% or if the minor allele frequency (MAF) was less than 1% across the whole data. The curated dataset for genomic analyses consisted of 48,062 SNPs mapped to the reference genome. Finally, using available dam genotypes (not presented), 161 markers (0.3% of curated markers) were excluded for having frequent opposite homozygotic genotypes in parent‐offspring pairs. Final data for the fullsib families comprised 47,901 markers and demonstrated a genotyping completeness of 99.7%. Eight samples were excluded based on sample genotype inconsistency suggesting mislabeling, leaving 279 individuals in the final data (Saimaa 95, BC_Neva_ 92, BC_Tornio_ 92).

### 2.4 Data Analyses

#### 2.4.1 Comparison of Crosses for Survival and Growth

Cross type differences in survival was tested using a generalized linear mixed model in SAS 9.4 (GLIMMIX procedure; SAS Institute, Cary, NC, USA), with logit link function and residual pseudo likelihood (RSPL) estimation technique. Here, the individual response was binary (0 = dead, 1 = alive at the end of the experiment). The model included crossing group as a fixed factor, whereas random intercepts were set for dams (i.e., covariation among maternal half sibs) as well as for experimental channels and rearing boxes, nested within a channel. When statistically testing for the main cross type effect, the channels, boxes and dams are assumed to be randomly drawn from a broader set of possible cases, and hence they are treated as random factors.

Cross type differences in fry body mass means were tested using a linear mixed model (MIXED procedure) with restricted maximum likelihood estimation method. The model included the same terms as that of survival, and further a fixed factor (’infdead’) took into account whether the observation was recorded from a live fish or if it had died during the preceding night due to experimental *Diplostomum* infection (see above). Thus, the additional term accounted for the short‐term increase of body mass due to osmotic absorption of water occurring between death and body mass measurement. Further, the potential effect of measurement time on body weight was accounted for by including cumulative water temperature sum from the start of the experiment until the date of measurement as a fixed covariate (‘Tsum’, range 399.1–426.3 degree‐days).

Standard errors and degrees of freedom for the tests of the fixed effects were calculated using the method by Kenward and Roger (1997). After the statistically significant overall test for the group means, their pairwise differences (least square means) were compared using Tukey–Kramer's adjustment.

#### 2.4.2 Comparison of Sire Performance From Different Crosses

To quantify the performance level of each sire from different crosses, a quantitative genetic analysis was performed across data from all crosses. To estimate genetic merit of sires for survival and final body mass, an animal model was used in which the observed variation in the traits was partitioned into the effect of pedigree, representing the genetic effect, permanent environmental effects of full sibs, residual variation, as well as systematic fixed factors. The variance components and genetic values of sires were estimated in DMU 6.0 software using a bivariate linear animal model and restricted maximum likelihood method (REML) (Madsen and Jensen 2008). The generalized linear model (GLMM) for binary survival was:

(1) $$y_{\mathrm{ijk}}=\mu +{\text{channel}}_{j}+{\text{anim}}_{i}+{\text{channel}}_{j}\times {\mathrm{box}}_{k}+e_{\mathrm{ijk}},$$

where μ is the model intercept, channel_*j*_ is the fixed effect of the experimental channel (*j* = 1 to 8), anim_*i*_ is the random genetic effect of an individual *i* associated with parental information (pedigree file), channel_*j*_ × box_*k*_ is the random interaction of the experimental channel and rearing box accounting for the permanent environment effect of full sibs, which is part of the total phenotypic variance to be estimated, and *e*_*ijk*_ is the residual of an observation *y*_*ijk*_. Treating channel as fixed in animal models eliminates the potential and confounding environmental heterogeneity among the channels.

For body mass, genetic parameters were estimated with the following model:

(2) $$y_{\mathrm{ijk}}=\mu +{\text{channel}}_{j}+{\text{infdead}}_{l}+\text{Tsum}+{\text{anim}}_{i}+{\text{channel}}_{j}\times {\mathrm{box}}_{k}+e_{\text{ijkl}}$$

Such models result in the estimates of genetic variance (*V*_*g*_), environment variance common to full sibs (*V*_*pe*_), and residual variance (*V*_*r*_) as well as best linear unbiased predictions (BLUP) of all animals in the pedigree, including all the sires. It should be noted that the variance components present variation across all the crosses. Heritability was calculated as $h^{2}=V_{g}\times {\left(V_{g}+V_{\mathrm{pe}}+V_{r}\right)}^{-1}$ and permanent environment effect was calculated as $c^{2}=V_{\mathrm{pe}}\times {\left(V_{g}+V_{\mathrm{pe}}+V_{r}\right)}^{-1}$. For binary survival (0 and 1 data), these variance ratios were further translated into the underlying liability scale following Dempster and Lerner (1950).

Using single‐generation phenotypic data, where body mass was only determined for individuals that survived until the end of the experiment, genetic correlation between body mass and survival could not be reliably estimated using the animal model. Therefore, the association between the two traits was explored by calculating sire‐family mean Spearman correlations between the traits using sires' BLUP from univariate animal models. A sire‐mean correlation gives an approximation of the underlying genetic correlation (Roff 1997; Astles et al. 2006). Further, for both traits, the BLUP means of sires from different strains (purebred and two hybrids, *n* = 18 for each cross) calculated using Models (1 and 2) were compared using analysis of variance (ANOVA) in a general linear model in SAS, with sire group as a fixed factor. After the significant ANOVA, pairwise comparisons of three groups of sire means were performed using Tukey–Kramer's adjustment of *p*‐values. Statistical significance was determined with a threshold value *α* = 0.05.

#### 2.4.3 Comparison of Genetic Diversity Among Crosses

To describe and compare the overall genomic diversity among the three crossing groups, summary statistics—directly observed polymorphism, minor allele frequency (MAF), expected heterozygosity (*H*_S_), and within population fixation index (F_IS_)—were calculated using standard functions of *R v4.4.1* (R Core Team 2024) and *dartR v2.9.9.5* (Mijangos et al. 2022). To quantify the partitioning of genetic variation among and within the groups, an analysis of molecular variance (AMOVA; Excoffier et al. 1992) was performed with *poppr.amova* in the package *poppr v2.9.8* (Kamvar et al. 2014), applying the *ade4* method implemented in *ade4 v1.7–23* (Dray and Dufour 2007). Population structure was further visualized and confirmed using principal component analysis (PCA) conducted with *snpgdsPCA* in *SNPRelate v1.38.1* (Zheng et al. 2012) with the exact algorithm and Bayesian normalization. Because the first principal component (PC1) clearly separated the pure Saimaa salmon from the two backcross groups, Saimaa ancestry was approximated by scaling individual PC1 scores so that the mean of the pure Saimaa salmon equaled 1 and the mean of the backcross groups equaled 0.75, corresponding to their expected ancestry proportions.

#### 2.4.4 Characterization of Homozygous Genomic Segments

To evaluate genomic regions in which backcrossing particularly influenced genomic inbreeding, the distribution of homozygosity‐by‐descent (HBD) segments, a probabilistic analogue of runs of homozygosity (ROH), was analyzed using *RZooRoH v0.4.1* (Bertrand et al. 2019). The conceptual framework follows Ceballos et al. (2018) and Kanaka et al. (2023). Unlike window‐based ROH methods, the Hidden Markov Model (HMM) approach in *RZooRoH* assigns posterior probabilities to genomic intervals and classifies them into HBD segments of different coalescence‐age depths, offering improved segment detection especially in data sets with modest SNP density and segments from different ancestral generations.

Estimation of HBD segments yielded individual inbreeding coefficients and effective population sizes (*N*ₑ). The HBD were inferred using the interval model, which explicitly assigns inferred segments to contiguous generation intervals forming generation classes. The interval model included three coalescent‐age classes in the HBD model corresponding to recent (generations 1–9), intermediate (10–18), and ancient (19–72) coalescence; however, these estimates do not represent precise demographic time points. With the typical generation interval of c. 5–6 years the recent ancestry approximately traces back to the initiation of the current captive breeding program 10 generations ago (Hutchings et al. 2019). Individual inbreeding coefficients were estimated from the posterior probabilities across these classes, and *N*_e_, representing idealized population producing comparable inbreeding, was inferred from each individual's inbreeding history. For each crossing type, *N*ₑ was summarized as the harmonic mean of full‐sib family estimates, with each family's estimate represented by its own harmonic mean across individuals. The main reason for the HBD analysis was to estimate inbreeding when ascertainment bias is a possible confounder and for the *N*ₑ analysis was to compare *N*ₑ estimate of recent Saimaa salmon in captive breeding to the same during wild reproduction period. The estimates for backcrosses are by‐products of genomic autozygosity estimation. Unlike most historical *N*ₑ estimators, *RZooRoH* is based on individual genomes rather than population samples and is designed to work also with sparse marker data. While one generation coalescence classes can be fitted, broader classes are more robust. For comparison, we further calculated the N_e_ estimates for Saimaa salmon using *currentNe2* (Santiago et al. 2025), utilizing the default recombination intensity and the observed full‐sib number.

Differences among the cross groups in the most recent HBD class occurrence at genome locations were assessed with a permutation‐based test. For each SNP, sample labels were permuted to generate a null distribution of log_2_ odds ratios (log_2_OR), and empirical *p*‐values were calculated as the proportion of permuted values exceeding the observed statistic using absolute values. The initial number of permutations used was 5000, and this was increased to 20,000 for SNPs reaching the minimum *p*‐value threshold to refine significance estimates. False discovery rate correction (method: Benjamini and Hochberg 1995) was applied across all SNPs to identify loci showing significant deviations.

## 3 Results

### 3.1 Survival From Fertilization to Late Alevin Stage

There were no significant differences in survival from fertilization to the end of the yolk sac stage between the different crossing groups: the mean was 51.5% for pure Saimaa salmon (SD 28.1%, range 0.0%–93.1%, *n* = 19 families), 55.5% for the BC_Neva_ group (SD 27.0%, range 0.0%–89.7%, *n* = 19 families), and 55.4% for the BC_Tornio_ (SD 27.9%, range 0.0%–89.0%, *n* = 19 families).

### 3.2 Phenotypic Comparison Among Purebred and Backcrossed Progeny at Fry Stage

After 1 month of semi‐natural growing, salmon cross types differed statistically significantly in their phenotypic mean survival (*F*_2,80.36_ = 3.57, *p* = 0.033). Pairwise comparisons showed that pure Saimaa salmon families survived significantly worse (least square mean ± SE = 0.669 ± 0.062) than their Tornio backcross half‐sib families (0.784 ± 0.049; Tukey‐adjusted *p* = 0.037), and also tended to survive worse than the Neva backcross families (0.763 ± 0.051; adjusted *p* = 0.117).

There were no statistically significant differences among cross types in fry body mass means (*F*_2,83.5_ = 0.14, *p* = 0.866). The model‐corrected mean ± SE was 2.28 ± 0.10 g for pure Saimaa salmon, 2.27 ± 0.10 g for Neva backcross, and 2.23 ± 0.10 g for Tornio backcross.

### 3.3 Quantitative Genetic Evaluation for Progeny Traits

Estimates of genetic variance, common‐environment variance, and their associated variance ratios are given in Table 1. Survival showed a moderate heritability of 0.307, whereas the common environment effect was slightly lower (*c* ^2^ = 0.183). For fry body mass, heritability (0.478) and the common environment effect (0.425) were also moderate and of similar magnitude.

**TABLE 1:** Number of data records, trait means, variance components^a^ (±SE) and variance ratios^b^ (±SE) for salmon fry survival and body mass estimated by a bivariate linear model.

	Survival	Body mass
*n*	2159	644
Mean (SD), g	0.704 (0.457)	2.203 (0.539)
*V*_*g*_	0.035 (0.013)	0.134 (0.055)
*V*_*pe*_	0.021 (0.005)	0.119 (0.024)
*V*_*r*_	0.144 (0.008)	0.027 (0.028)
*h* ^2^	0.307 (0.079)^c^	0.478 (0.118)
*c* ^2^	0.183 (0.035)^c^	0.425 (0.078)

^a^ *V*_*g*_ = additive genetic variance; *V*_*pe*_ = permanent environment variance due to dams (survival) or full‐sib groups (body mass); *V*_*r*_ = residual variance.

^b^ Heritability—$h^{2}=V_{g}\times {\left(V_{g}+V_{\mathrm{pe}}+V_{r}\right)}^{-1}$; common environment effect ratio—$c^{2}=V_{\mathrm{pe}}\times {\left(V_{g}+V_{\mathrm{pe}}+V_{r}\right)}^{-1}$.

^c^ Estimate ± SE calculated on the liability scale following Dempster and Lerner (1950).

Correlation of genetic values (BLUPs) of survival and body mass among sires was positive and marginally significant (Pearson's *r* = 0.262, *p* = 0.056, *n* = 54 sires), indicating a weak tendency for genetically heavier families to also survive better.

There was a statistically significant genetic difference in sire‐mean BLUPs of survival among sire groups (*F*_2,51_ = 4.09, *p* = 0.023). Purebred Saimaa sires had, on average, lower genetic merit for survival (mean = −0.098) than hybrid sires. The difference was significant in relation to Saimaa‐Tornio hybrids (mean = 0.062, *p* = 0.027) and close to significance in relation to Saimaa‐Neva hybrids (mean = 0.035, *p* = 0.077). The two hybrid sire groups did not differ significantly (*p* = 0.895).

For fry body mass, the mean genetic merits did not statistically differ among the three sire groups (*F*_2,51_ = 0.07, *p* = 0.934). These sire‐level comparisons align well with the phenotypic results, which likewise showed higher survival in the backcrosses but no cross‐type differences in body size.

### 3.4 Population Structure and Admixture Confirmation

AMOVA divided each maternal half‐sib family into three full‐sib family types. Most of the genetic differentiation occurred among the backcross types (Φ = 0.16), with comparatively modest differences among half‐sib families overall (Φ = 0.061) and no detectable subdivision within full‐sib families (Φ slightly negative). This confirmed the backcross type as the main cause of genetic differentiation, as expected.

In PCA, Saimaa salmon vs. backcrosses formed the main division (Figure 2). The first principal component explained 6.0% of the total genomic variation and clearly separated pure Saimaa salmon from both backcross types, while the second component explained 3.0% of the variation and distinguished the two backcross types from each other. Saimaa salmon clustered more tightly than either of the crossbred groups. There was no visible clustering based on half‐sib family structure, and the clustering according to full‐sib family groups was clearly less prominent (more compact) than clustering according to the backcross types. Together, these results confirm that backcross type is the dominant source of genetic structure, show no signs of group‐level mislabeling, and indicate relative genetic homogeneity within the Saimaa salmon group compared with the greater variation observed among backcross individuals.

**FIGURE 2:**
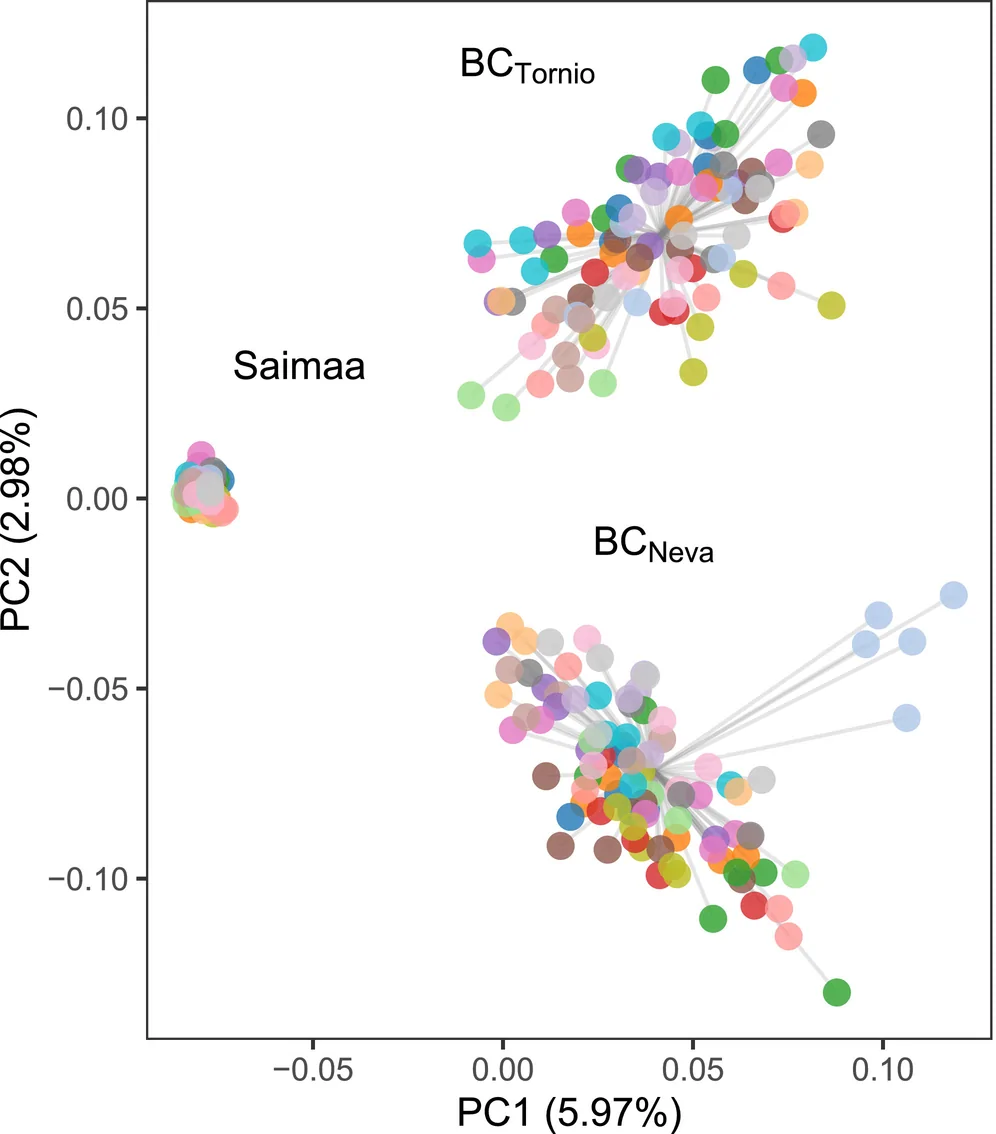
A PCA plot illustrating both the dominating effect of different introgressed genetic alleles and extensive within‐group variation. The points are colored based on dam, and the visible clustering of similar colors indicates full‐sib family clusters. Radial lines connect each individual to the centroid of its backcross group.

### 3.5 Genomic Diversity

When treating the cross types as separate populations, their differentiation (*F*_ST_) was low (0.029). As expected for a backcross design, the within‐type inbreeding coefficient was negative (*F*_IS_ = −0.092), reflecting lower than random relatedness between the parents in the mating design. Mean genomic diversity was lowest in pure Saimaa salmon, compared to both Tornio and Neva backcrosses (Table 2). Of the 47,901 SNPs included, 56% were monomorphic in Saimaa salmon, where the corresponding proportions were much lower in the backcrosses (3%–4%). Kolmogorov–Smirnov tests indicated that all population types differed significantly (*p* < 0.001) in the proportions of polymorphic loci, including the Tornio and Neva backcrosses, which showed the smallest difference (1.0%, or 465 loci). For average minor allele frequency (MAF) and within‐population genetic diversity (*H*_S_), no significant differences were observed between Tornio and Neva backcrosses, whereas both were substantially more variable than the pure Saimaa salmon (Table 2).

**TABLE 2:** Population diversity metrics with 95% confidence intervals shown in parentheses or across family standard errors for effective population sizes (*N*_e_) and standard deviation for total accumulated inbreeding.

Group	Variable loci	Non‐variable proportion	MAF (all loci)	MAF (variable loci)	Private alleles	Absent alleles	*H*_S_ (95% CI)	*F*_IS_ (95% CI)	*N*_e recent_ (95% CI)	*N*_e old_ (95% CI)	Inbreeding (SD)
Saimaa salmon	21,221	0.557	0.098	0.220	4	26,680	0.133 (0.078–0.149)	−0.025 (−0.015–0.038)	80 (70–91)	45 (45–46)	0.48 (0.03)
Neva backcross	45,858	0.043	0.171	0.179	1520	2043	0.248 (0.204–0.262)	−0.096 (−0.122– −0.086)	91 (75–112)	146 (137–159)	0.23 (0.05)
Tornio backcross	46,323	0.033	0.172	0.178	1962	1578	0.250 (0.217–0.274)	−0.090 (−0.123– −0.086)	88 (76–107)	139 (130–148)	0.24 (0.05)

*Note:* Absent alleles are external private alleles, not observed in the focal population (cross type). *N*_e_ was calculated for recent generations and for old natural endangerment situation (prior to transition to captive breeding).

Abbreviations: *F*_IS_, within‐type inbreeding coefficient; *H*_S_, within‐type genetic diversity; MAF, minor allele frequency.

Both Tornio and Neva backcross types possessed many polymorphic loci that were not variable in Saimaa salmon (25,366 and 24,986 loci, respectively). They also each retained private polymorphisms relative to one another (1962 unique to BC_Tornio_ and 1520 to BC_Neva_). In contrast, only a small number of loci (4) were variable in Saimaa salmon only. Saimaa salmon polymorphisms that were monomorphic in one of the two backcrosses were a bit more common (58 for BC_Tornio_, 81 for BC_Neva_). The Tornio and Neva backcrosses had 2039 and 1574 variable loci that were fixed in the other backcross type, respectively. Therefore, each backcross showed a distinct but minor advantage, BC_Tornio_, in retaining Saimaa‐origin variants, and BC_Neva_ in introducing fewer donor‐specific alleles. When considering only variable loci, Saimaa salmon average MAF (0.220) exceeds that of the backcrosses (0.178 and 0.179), as the introduced variants remain rare causing an excess of loci with small MAF in backcross populations (Figure 3).

**FIGURE 3:**
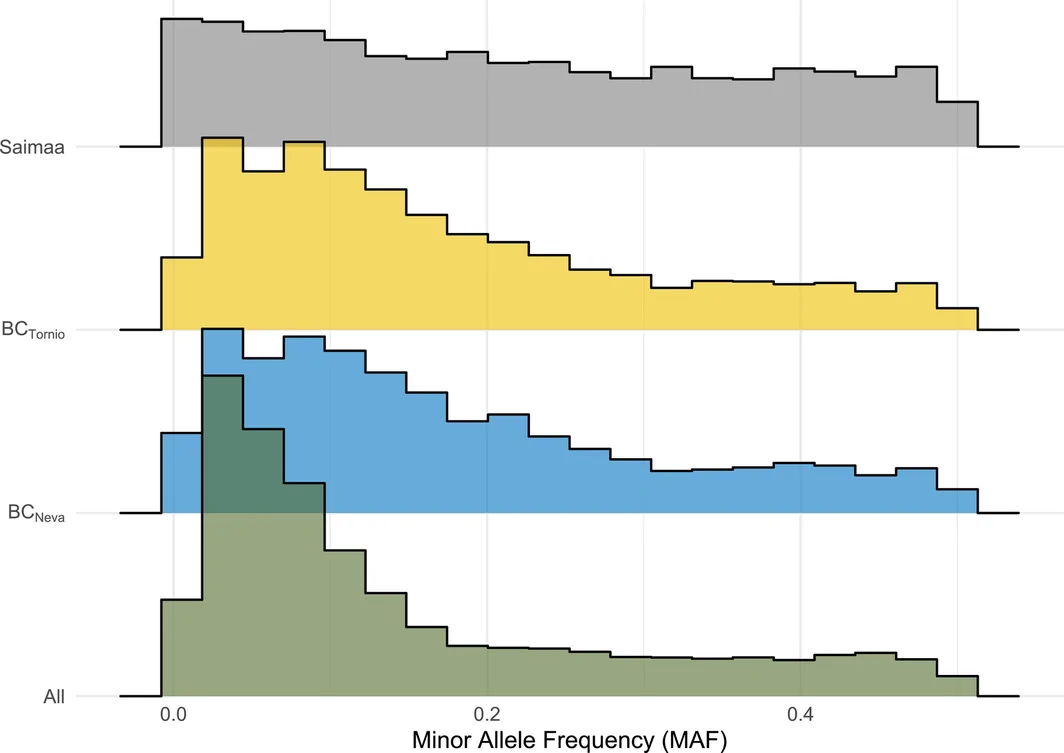
Minor allele frequency (MAF) histograms for different backcross types and the combined population indicating backcrossing increases the share of loci with low MAF values.

The proportion of Saimaa salmon ancestry in an individual (derived from PC1 score) was negatively associated with observed heterozygosity (regression coefficient −0.55, *p* < 0.001, *n* = 279; Figure 4), indicating that individuals with higher Saimaa ancestry had fewer heterozygous loci. Across the observed ancestry range (0.55–1.00), heterozygosity declined linearly from 0.38 to 0.18, with the predicted value at 0.75 corresponding closely to the mean observed in the backcrosses (0.27). Sixteen individuals, scattered across dams, showed elevated heterozygosity relative to the regression trend (Figure 4). These outliers had more missing data at low‐variability loci, but their pattern was unrelated to Saimaa ancestry and had negligible impact on overall population estimates. Observed heterozygosity can be used as a practical proxy for Saimaa ancestry (~(0.68‐H_O_) × 0.55^−1^), with regression residuals indicating only minor error (a few percentage points) relative to PCA‐based estimates.

**FIGURE 4:**
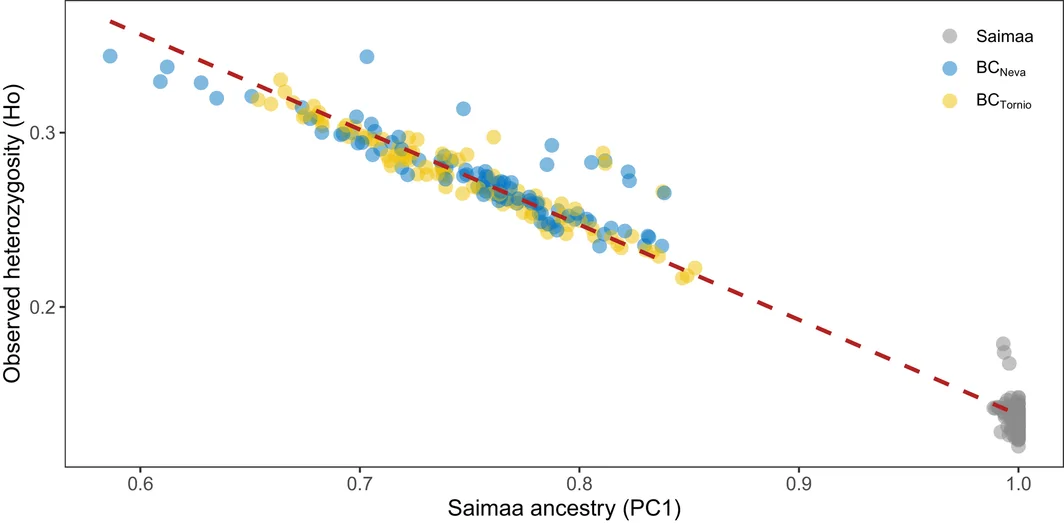
Observed heterozygosity (H_O_) regressed against the proportion of Saimaa salmon ancestry (*n* = 279 fish). The salmon cross types are purebred Saimaa salmon and their two backcrossed half‐sibs (BC_Neva_ and BC_Tornio_).

### 3.6 Runs of Homozygosity

The effective population size (*N*_e_) estimates for the three groups ranged from 80 to 91, with slightly higher values in the backcrosses (Table 2). For pure Saimaa salmon, the recent *N*_e_ estimates were nearly twice the historical values, indicating that the conservation breeding program has slowed down the accumulation of inbreeding compared with the wild populations before the initiation of captive breeding. While the HBD derived captive‐period *N*_e_ estimate of 80 is very similar to *N*_e_ estimate of 82 obtained from *currentNe2* using all marker pairs, it may be biased upwards by the early captive generations, and the more recent captive size might be closer to 50 (results not shown). The low ancient *N*_e_ estimate for Saimaa salmon (45) reflects demographic conditions prior to conservation action. However, this genetic bottleneck might be close to the ancient limit of the current HBD coalescence simulation rather than very recent.

Backcross groups showed only moderately increased recent *N*_e_ while backcrossing roughly halved the realized genomic inbreeding relative to pure Saimaa salmon (Table 2). In general, overall genomic inbreeding estimates were stable against varying the population model while *N*_e_ assigning age to this inbreeding was more variable. However, tracing the differences in inbreeding to differences in the separate HBD age classes (detailed frequencies not shown) indicated that the reduction in autozygosity in the backcrosses was driven primarily by the loss of dominant short, ancient ROH. Saimaa salmon genomes were dominated by the deepest HBD age class (~41% of the genome), whereas this component declined to 16% in both backcross groups, as introgressed genomic regions reduce the proportion of the genome assigned to the historical low *N*_e_ coalescent background. In contrast, the proportion of long recent HBD remained similar across populations (5%–6%), being highest in Saimaa salmon. The combined recent and intermediate HBD in the backcrosses (7%–8%) approximates the recent component alone in Saimaa salmon, indicating that backcrossing primarily reduces the dominant ancient autozygosity (Table 2), as a smaller proportion of the genome is assigned to coalescence events within the modeled genealogy.

Although the single‐population interval model did not fit the backcross populations as well as it did for the pure Saimaa salmon (likelihoods not shown) and in backcrosses non‐convergence begins to interfere if including more ancient generations than here, the estimation nonetheless provided the best available representation of the observed accumulation of HBD segments in this dataset. The resulting estimates therefore offer a neutral basis for comparing inbreeding across the three crossing types but the *N*_e_ estimates using the same time windows for all populations should not be considered demographic parameters of real populations.

### 3.7 Identifying of Genomic Regions Influenced by Backcrossing

Assessment of changes in recent HBD occurrence at individual SNP positions following admixture revealed widespread genomic effects of backcrossing (Figure 5). Of the 47,901 SNPs analyzed, 22,112 showed a reduction in recent HBD in both backcross groups, whereas 9222 showed an increase in both groups. The remaining 16,567 SNPs exhibited backcross‐specific directions of change. Overall, 9755 SNPs (20.4%) were located in genomic regions where the frequency of recent HBD segments differed significantly between pure Saimaa salmon and at least one of the two backcross groups (Figure 5). Among these, 2821 SNPs displayed significant deviations in both backcross comparisons, and these deviations were almost exclusively positive (99.7%), indicating a consistent reduction of recent HBD in the backcrosses relative to the pure Saimaa salmon. Despite significant differences, which filter out family‐specific patterns, the genomic impact of admixture on recent autozygosity was highly concordant between the two backcross groups.

**FIGURE 5:**
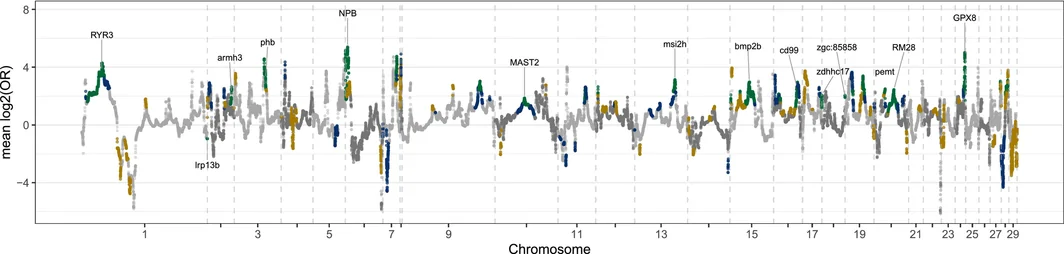
Manhattan plot of HBD segment probability differences across chromosomes (*x* axis). Points represent mean log_2_ odds ratios for genomic regions colors indicating significance from FDR‐corrected permutation tests at 5% level: Gray = non‐significant, yellow = significant deviation in Tornio backcross comparison, blue = significant deviation in Neva backcross comparison, green = significant deviation in both comparisons between pure and backcrosses. Labels identify candidate genes in the main deviating regions (maximum one per chromosome), and the only consistently negative region resisting introgression is annotated with lrp13b. Positive log_2_(OR) values indicate regions consistently more inbred in pure Saimaa salmon and thus favoring introgression (i.e., a SNP is more frequently in an HBD segment in Saimaa salmon; value 1 means that Saimaa Salmon has twice the odds), while negative values indicate regions more inbred in backcross populations and therefore resisting introgression.

Only eight SNPs, all clustered at the end of chromosome SSA1 (around 174 Mb), showed the pattern where they were situated in more inbred segments in both backcross types. This region therefore represents the single consistent exception in which Saimaa salmon were less inbred than the backcrosses, suggesting either a localized inheritance pattern or selection.

## 4 Discussion

In this study, we provide empirical evidence that assisted gene flow can offer a potential viability advantage for the small and highly inbred landlocked Saimaa salmon population at a critical early life stage when natural mortality is typically very high. Supporting earlier expectations for the hybridization benefits at both phenotypic and genetic levels, the backcrossed first‐feeding fry survived better than their purebred half‐siblings under controlled semi‐natural stream conditions. Genomic analysis further showed that backcrossed salmon progeny exhibited markedly higher heterozygosity and a much lower proportion of their genome contained inbred segments (ROH) compared with pure Saimaa salmon. Although introgression was not uniformly distributed across the genome, most affected regions showed reduced homozygosity in the pure Saimaa salmon population, indicating that both donor lineages successfully introduced new allelic variation.

Taken together, these findings demonstrate that introducing approximately one‐quarter of exotic ancestry through a maternal backcross can substantially alleviate the extreme genomic fixation and inbreeding burden characteristic of Saimaa salmon. At the same time, we recognize that the present study captures only a single generation, and that early backcrosses carry introgressed alleles in heterozygous form; thus, any recessive or epistatic incompatibilities may remain undetected at this stage (Edmands 1999, 2007; Hedrick and Garcia‐Dorado 2016). Our results therefore provide an important early indication of genetic‐rescue potential, while underscoring the need for continued multi‐generation monitoring of phenotypic performance, genomic integrity, and the possible emergence of deleterious interactions as hybridization progresses.

### 4.1 Phenotypic and Genetic Evaluation of Cross Types

Survival of fry showed clear differences (9.4–11.5 percentage‐point) between purebred and backcrossed families, evident both phenotypically and at the genetic sire level. Although the observed survival advantage of the backcrosses relative to pure Saimaa salmon may be partly consistent with additive gene action, it is equally compatible with partial dominance or overdominance (heterosis), reflecting the masking of deleterious recessive alleles in the inbred Saimaa lineage (Roff 2002). In a similar experimental setting, Eronen et al. (2021) also reported a potential heterosis effect in survival of F1‐generation fry when Tornio and Neva females were crossed with Saimaa males, although maternal effects probably played a role in the results, too. Even though the manifestation of inbreeding depression is commonplace, it depends on several factors, including environmental conditions and the trait(s) studied (Armbruster and Reed 2005; Hedrick and Garcia‐Dorado 2016). In our case, the test environment imposed substantial viability stress due to the limited availability of natural food. This fundamentally differs from the conventional hatchery‐rearing conditions used for the production of Saimaa salmon; that is, plain plastic or fiberglass tanks, where dense masses of fry are constantly fed with commercial dry pellets. Survival during the critical onset of external feeding is known to be partly size‐selective and sensitive to maternal influences as well as genetic factors (Hutchings 1991; Einum and Fleming 1999, 2000a, 2000b; Einum 2003; Janhunen et al. 2024). Such selective mortality across the crossing groups might explain why we could not detect differences in fry body mass means, despite the high heritability of the trait (0.48). It is also possible that growth was partly density‐dependent at the phenotypic level; that is, in experimental units where mortality was high, competition among the remaining siblings was lower and growth, therefore, higher (raw correlation of survival and body weight means: Pearson's *r* = 0.354, *p* < 0.001, *n* = 106 rearing boxes).

Importantly, intrinsic outbreeding depression often appears in the second (F2) or later outbred generations, when full recombination of parental genomes occurs, causing dominant incompatibility or the breakdown of locally adapted gene complexes in the first backcrosses (Edmands 2007). Despite the long evolutionary divergence of Saimaa salmon from the two anadromous populations, however, we did not observe measurable phenotypic signs of outbreeding depression during incubation (from fertilization to alevin stage) or during the critical transition period from endogenous to exogenous feeding. This conforms to the general view that introducing novel alleles from outcrossing can be beneficial for an inbred population, often outweighing the risks of outbreeding depression (McClelland and Naish 2007; Frankham 2015; Ralls et al. 2020; Pregler et al. 2023). Furthermore, considering that both Neva and Tornio grandparents also represented hatchery strains (first hatchery generation) and Neva population even has a long history in captive breeding (see Eronen et al. 2023), backcrossing might have resulted in partial disruption of hatchery‐adaptive alleles from their sides as well. Either way, as the present genomic results show, the genetic interaction (introgression) between population pairs is inconsistent and difficult to predict; this underlines the need for evaluating outbreeding effects on a case‐by‐case basis.

Hybridization effects beyond the F1 generation have been examined in several salmonid systems (e.g., McClelland et al. 2005; Vandersteen et al. 2012; Lehnert et al. 2014; Pregler et al. 2023). Unlike our finding, some studies report reduced fitness and adaptation in backcross generations. For example, in crosses between wild and farmed Atlantic salmon, outbreeding depression persisted beyond the F1 generation, and both demographic (survival) and molecular (gene expression) signs remained detectable in later generations (McGinnity et al. 2003; Roberge et al. 2008). Backcrossing did not eliminate the maladaptive domestication effects, and transcriptional divergence was even greater than that observed between pure wild and farmed salmon (Roberge et al. 2008). Similarly reduced marine survival in F1 and F2 hybrids of allopatric populations of pink salmon ( *Oncorhynchus gorbuscha* ) was interpreted as outbreeding depression (Gilk et al. 2004). In a follow‐up study, Wang et al. (2007) suggested that this decline in hybrid fitness was likely attributable to mismatched developmental timing at hatching, reflecting local thermal adaptation in their original rivers. Given that our present study exclusively focuses on the fry stage, the possibility for outbreeding depression still prevails later in life. Although the life histories of both landlocked and anadromous salmon do share early freshwater residency in river habitats, they substantially diverge in terms of geographical river conditions (e.g., regional thermal regimes) and migration environments (i.e., structurally complex lakes vs. wide open sea).

Both inbreeding and outbreeding effects can manifest in highly variable ways, depending on the life stage and trait studied, the populations being assessed, and the environmental context (Tymchuk et al. 2007; Paul et al. 2022). In our study, we intended to mimic natural stream conditions because there is an on‐going conservation program with the primary goal to increase natural reproduction in restored river habitats downstream and upstream of hydroelectric dams (Leinonen et al. 2020). Houde et al. (2011a, 2011b) crossbred three geographically close Atlantic salmon populations and compared early survival and growth of hybrids with purebred and inbred lines over two generations both under hatchery and natural conditions. While no clear signs of inbreeding depression, heterosis, nor outbreeding depression were detected in captive rearing (Houde et al. 2011a), a trend towards reduced survival in one hybrid strain emerged in the natural environment, possibly indicating a loss of local adaptation (Houde et al. 2011b).

### 4.2 Genomic Diversity and ROHs

Genome‐wide SNP information demonstrated the very strong impact of crossbreeding and deepens earlier perception based on a few microsatellite and allozyme data that Saimaa salmon are suffering from a severe reduction of genetic diversity due to historical population bottlenecks (Vuorinen 1982; Koljonen et al. 2002; Säisä et al. 2005; Tonteri et al. 2005). The backcross families possessed approximately twice the number of polymorphic loci and nearly double the heterozygosity (0.25 vs. 0.13) compared to their purebred Saimaa salmon half‐siblings. In total, the backcrosses gained 3482 alleles, whereas only four alleles were unique to the pure Saimaa salmon. In our case, a simple mixture model helps to illustrate the sources of increased gene diversity in the first backcross (BC_1_) generation. Using the approximation *H*_S(BC₁)_ ≈ (3/4) *H*_S(SaS)_ + (1/4) *H*_S(donor)_ + (3/4) *D*_ST_, and inserting the observed values (*H*_S(SaS)_ ≈ 0.13, *H*_S(BC₁)_ ≈ 0.25, *G*_ST(SaS vs. BC₁)_ ≈ 0.03) under the fixed backcross mating scheme, we estimate that the BC_1_ heterozygosity is derived roughly 45% from Saimaa salmon diversity, 40% from donor diversity, and about 15% from between‐population differentiation (*H*_S(donor)_ ≈ 0.47, *D*_ST (SaS vs. donor)_ ≈ 0.05) (Nei 1973). While these values help interpreting the BC_1_ by emphasizing the impact of diversity in the donor population, these values should be treated cautiously, as the approximation does not account for structural variation, sex‐linked inheritance, or selection during early development.

Our analyses of runs of homozygosity (ROH), that is, homozygosity‐by‐descent (HBD) segments inferred by the RZooRoH model, further support the view that pure Saimaa salmon possess eroded genomic variation dominated by long‐term autozygosity, consistent with substantial historical genetic load. In contrast, the backcross populations displayed markedly higher genetic diversity (*H*_S_ ≈ 0.25 vs. 0.13 in Saimaa salmon) and a major reduction in genome‐wide ROH. Because the distribution and length of ROH reflect the cumulative effects of drift, bottlenecks, and inbreeding (McQuillan et al. 2008; MacLeod et al. 2013; Curik et al. 2014; Peripolli et al. 2017; Brüniche‐Olsen et al. 2018; Ceballos et al. 2018), these patterns provide insight into the accumulation of genomic autozygosity in the lineages.

In Saimaa salmon, most autozygosity was contained in short, deeper HBD model age classes rather than in long recent ROH, suggesting that the bulk of inbreeding accumulated prior to the initiation of the conservation program (1963–1969, i.e., approx. 10 generations ago). Following backcrossing, these ancient coalescing segments were substantially reduced, while the proportion of combined recent and intermediate (longer) HBD changed little. In general, aquaculture populations are prone to genetic erosion and inbreeding due to small effective population size and breeding management practices (Tave 1999; D'Ambrosio et al. 2019), but in Saimaa salmon the primary effect of backcrossing is the dilution of historical autozygosity rather than the removal of contemporary inbreeding. A majority (0.414 which is 87% of the total) of inbreeding in Saimaa salmon seems to have occurred before the initiation of conservation. It also suggests that recent captive breeding practices have maintained at least the pre‐conservation level effective population size even when it had to rely on captive‐born fish as parents. The introduction of donor alleles reshapes the genomic landscape by replacing some homozygous blocks with heterozygous segments, but without much influencing the total recent inbreeding.

Two earlier studies on Saimaa salmon have linked low genetic diversity (measured via microsatellites) to impaired foraging success and early developmental malformations (Primmer et al. 2003; Tiira et al. 2006). Some fish studies have examined genome‐wide ROH patterns in assessing genetic diversity, inbreeding and the effects of natural or artificial selection (D'Ambrosio et al. 2019; Aramburu et al. 2020; Yoshida et al. 2020; De los Ríos‐Pérez et al. 2022; Subramanian and Kumar 2023). To our knowledge, however, only a few of them have so far associated ROH metrics with measurable phenotypic effects (Paul et al. 2022; Lyu et al. 2026). Our results for early survival suggest that genomic inbreeding can have biologically meaningful effects on performance (fitness), consistent with similar findings across diverse vertebrates (reviewed by Kyriazis et al. 2025). However, the striking ROH differences between Saimaa salmon and the backcrosses did not translate into measurable differences in body mass among survivors, nor did individual heterozygosity correlate with growth (Pearson's *r* = −0.080, *p* = 0.183, *n* = 279 fish). One explanation may be that inbreeding depression occurs at specific genomic regions rather than reflecting uniform genome‐wide effects. Interestingly, Paul et al. (2022) showed in rainbow trout ( *Oncorhynchus mykiss* ) that long ROH (indicative of recent inbreeding events) were linked to negative effects on female spawn weight, egg number, and body size traits, while short ROH regions showed weaker or even positive effects—likely due to fixation of favorable alleles or overdominance effects. Findings like this highlight the complex architecture of inbreeding depression beyond average genome‐wide measures; they also call for further investigation in Saimaa salmon to locate the most relevant ROH underlying inbreeding and crossing impacts on performance traits.

### 4.3 Genome‐Wide Introgression

At a broad level, admixture converted many previously monomorphic genomic regions in Saimaa salmon into low‐frequency polymorphisms, as expected when divergent allele pools are combined. Because recent (long) ROH are often most relevant to fitness effects (e.g., Martikainen et al. 2020; Ablondi et al. 2023; Swinford et al. 2023), we focused on how backcrossing altered their genomic distribution. We detected widespread but heterogeneous changes: roughly one‐fifth of SNPs lay in regions where the frequency of recent HBD differed between Saimaa salmon and at least one backcross, yet only about 6% of all loci (~29% of significant sites) showed a shared reduction in recent HBD across both backcrosses. This indicates that many genomic segments respond similarly to admixture, but a substantial fraction is donor‐specific and, in some cases, family‐dependent (i.e., contingent on particular dam × F1‐sire combinations), consistent with source‐ or target‐family‐dependent introgression dynamics.

Overall, the observed patterns of introgression suggest non‐uniformity between the cross types, warranting further studies. However, functional interpretation should remain cautious. Reduced inbreeding in some regions likely reflects the introduction of novel alleles into previously homozygous blocks, but the changes were not consistently replicated between backcrosses. Further, these novel alleles introduced by outbreeding do not, by themselves, demonstrate selection. Under neutral expectations, introgression should break up long Saimaa autozygous segments by introducing donor haplotypes into previously identical‐by‐descent regions, increasing local heterozygosity and reducing the frequency of recent HBD. Most observed local decreases followed this pattern, and their inconsistent appearance across the two backcrosses is compatible with neutral segregation, recombination, and family‐specific haplotype contributions. A smaller subset of loci showed consistent reductions in both backcrosses, reflecting regions where backcrossing consistently increased heterozygosity. In contrast, consistent deviations from the expectation that backcrosses show less genomic inbreeding than pure Saimaa salmon were very rare. The only clear case was a small interval at the distal end of SSA1, containing eight SNPs, that resisted introgression in both cross types. This region maps to lrp13b, sometimes annotated as lrp2, a gene essential for yolk formation and female fertility in teleosts (Liu et al. 2024), making it a plausible candidate for localized selection or inheritance bias. Taken together, these patterns indicate that while most of the genome follows neutral introgression dynamics, a few regions deviate in ways that warrant targeted validation.

Three further caveats frame our interpretation. First, the progeny experienced only one survival‐selection episode at an early life stage, and longer‐term fitness components remain to be assessed. Second, since the SNP array used in our study was originally developed for commercially reared Atlantic salmon, it is not optimal for the genomic assessment of diversity in Saimaa salmon. This ascertainment bias likely affects minor allele frequency distributions, heterozygosity estimates, and the proportion of monomorphic loci. While the conclusion that Saimaa salmon has low diversity and backcrosses vary substantially more is undoubtedly correct, the magnitudes of genomic diversity metrics may be distorted. Genome‐wide estimates based on haplotypes and ROH are less sensitive to ascertainment bias, but genome reorganization may disturb estimates locally, warranting strain‐specific genome assemblies. Third, a broader genomic survey of the Saimaa hatchery broodstock across several year classes is needed to measure the current genomic architecture of the population. Even with these limitations, our half‐sib design provides a clear picture of the first‐generation change: backcrossing generates new variation int. the inbred genomic blocks of Saimaa salmon and reduces their inbreeding estimates by half.

### 4.4 Concluding Remarks

To our knowledge, the present study is the first to experimentally assess the fitness aspects of hybridization (first backcross) while also describing its impact on the genomic structure of a fish population, including specific changes occurring in inbred segments. Our finding that backcrossed progeny had higher early survival than pure Saimaa salmon is consistent with the presence of deleterious recessive alleles contributing to inbreeding depression in the Saimaa salmon population. This pattern supports the partial dominance hypothesis (i.e., masking of deleterious alleles via increased heterozygosity; Roff 2002; Paul et al. 2022), while not excluding contributions from additive foreign sire effects or overdominance (heterozygote advantage) as alternative or complementary explanations. In general, essential genes tend to be constrained by stronger purifying selection and thus are less variable in a population (Hedrick and Garcia‐Dorado 2016; Pérez‐Pereira et al. 2022). Long‐term management aimed at slowing inbreeding and drift in the Saimaa salmon may have limited further genomic fixation and purging. Notably, the captive broodstock has been renewed annually with wild‐caught spawners that completed the migratory phase in the wild (Janhunen et al. 2023), which could facilitate purifying selection against highly deleterious variants (Day et al. 2003; Hedrick and Garcia‐Dorado 2016). However, presence of detrimental alleles is still obvious, and very low genomic diversity and demographic stochasticity continue to threaten long‐term population health (e.g., Grossen et al. 2020; Kardos et al. 2021; Mathur and DeWoody 2021; Mathur et al. 2023). The present alevin‐fry results suggest that persistent recessive load is particularly expressed in early life traits that have been largely shielded from natural selection for multiple generations (nearly six decades). On the other hand, even if selection favors adaptive alleles introduced from foreign population(s), carefully managed, recurrent *ex‐situ* backcrossing increases the likelihood that Saimaa salmon population retains its important ancestral genetic variation and ecological identity (Bay et al. 2019; Wharton 2023; Graf 2024).

In summary, while adaptive differentiation between the landlocked and anadromous lineages should not be overlooked, our results highlight the short‐term fitness and genomic benefits that controlled backcrossing can provide by restoring depleted diversity and substantially reducing the burden of historical, genome‐wide inbreeding in Saimaa salmon. Although most autozygosity reflects ancient demographic events, our analysis also indicates that some recent inbreeding persists and will inevitably re‐accumulate under continued captive propagation. The inherited legacy of deep inbreeding and the ongoing risk of new inbreeding underscore the need for genetic reinforcement, which is supported by the observed improvement in early viability under semi‐natural conditions. At the same time, genetic rescue alone cannot restore a self‐sustaining population unless the fundamental ecological constraints, most critically the scarcity of natural spawning and nursery areas, are also addressed. Because Saimaa salmon management will necessarily continue to rely on *ex‐situ* breeding, integrating genomic monitoring into routine practice is essential, and post‐release assessments across life history stages will be required to ensure that local adaptation and population identity are retained in the hybridized strain.

## Funding

Funding was provided by the Ministry of Agriculture and Forestry of Finland (project: 4400T‐0714), Natural Resources Institute Finland (incl. its Statutory Services Unit, VOAS), the Raija and Ossi Tuuliainen Foundation, and the European Maritime and Fisheries Fund (project: 337793).

## Conflicts of Interest

The authors declare no conflicts of interest.

## Data Availability

The data and analysis scripts that support the findings of this study are openly available in Mendeley Data at https://data.mendeley.com/datasets/ws56v9rh77/1, doi:10.17632/ws56v9rh77.1.
